# Role of Haematopoietic Stem Cell Transplantation in Peripheral T-Cell Lymphoma

**DOI:** 10.3390/cancers12113125

**Published:** 2020-10-26

**Authors:** Chathuri Abeyakoon, Carrie van der Weyden, Sean Harrop, Amit Khot, Michael Dickinson, Costas K. Yannakou, H. Miles Prince

**Affiliations:** 1 Epworth HealthCare, Melbourne, Victoria 3002, Australia; sean.harrop@epworth.org.au (S.H.); Costas.Yannakou@epworth.org.au (C.K.Y.); Miles.Prince@petermac.org (H.M.P.); 2 Peter MacCallum Cancer Centre, Melbourne, Victoria 3000, Australia; Carrie.VanDerWeyden@petermac.org (C.v.d.W.); Amit.Khot@petermac.org (A.K.); Michael.Dickinson@petermac.org (M.D.); 3 Sir Peter MacCallum Department of Oncology, University of Melbourne, Victoria 3000, Australia; 4 Royal Melbourne Hospital, Melbourne, Victoria 3052, Australia

**Keywords:** peripheral T-cell lymphoma, PTCL, haematopoietic stem cell transplantation

## Abstract

**Simple Summary:**

The role of haematopoietic stem cell transplantation in the management of peripheral T-cell lymphomas is not clearly defined and therapeutic decisions vary widely across different institutions. This review examines the current body of evidence to best delineate the role and timing of transplantation in an era where diagnostic techniques and targeted therapies are rapidly evolving.

**Abstract:**

Peripheral T-cell lymphomas (PTCLs) are distinct pathological entities with clinical advancements lagging behind their B-cell lymphoma counterpart. Frequently aggressive in their clinical behaviour, clinicians are constantly challenged with low complete remission rates, early relapses and failure to achieve long-term responses despite aggressive first-line chemotherapy, resulting in poor overall survival in the majority of patients. There is currently no consensus regarding the optimal therapy for PTCL and treatment approaches are mainly derived from prospective phase II studies, registry data and retrospective studies. Despite its biological heterogeneity, a less than satisfactory “one-size-fits-all” approach has been adopted to date. Although its role remains controversial, for many years, haematopoietic stem cell transplantation has been adopted by clinicians with the aim of overcoming poor outcomes by consolidating responses. In this review, we aim to define the role of both autologous and allogeneic stem cell transplantation in PTCL in both frontline and salvage settings, especially in the context of recent advancements in this field.

## 1. Introduction

Due to the rarity and heterogeneity of peripheral T-cell lymphomas (PTCLs), few randomised prospective controlled trials currently exist to guide their management. Consequently, the management of PTCL still remains a therapeutic challenge. Despite the wide variation in clinical and biological behaviours of each PTCL subtype, management has largely taken a less than satisfactory “one-size-fits-all” approach, applying non-targeted therapy to a heterogeneous disease entity. To date, management approaches are primarily derived from phase II studies, retrospective series and clinical experience. 

Extrapolating from the management of B-cell non-Hodgkin lymphoma (B-NHL), anthracycline-containing regimens such as cyclophosphamide, doxorubicin, vincristine and prednisolone (CHOP) or CHOP-like regimens represent the cornerstone of frontline treatment for PTCLs. However, outcomes have been inferior with overall response rates for PTCL ranging from 50–70% compared with the more favourable overall response rates of 80–90% in B-NHL [1,2]. In addition to inferior overall response rates compared with B-NHL, PTCLs also have inferior complete remission (CR) rates, poor progression-free survival (PFS) and overall survival (OS), even in the more favourable anaplastic large-cell lymphoma (ALCL), anaplastic lymphoma kinase (ALK)-positive subtype [1]. A number of phase I and phase II trials have attempted to improve these results by intensifying treatment either with (i) alternative more aggressive chemotherapy regimens, (ii) the addition of a novel agent to a CHOP-backbone and/or (iii) consolidation with haematopoietic stem cell transplantation (HSCT). 

When evaluating the current available data, it is important to note that the majority of published trials have excluded patients who are ALCL, ALK-positive as these patients have a superior outcome with CHOP and CHOP-like regimens as opposed to other PTCL subtypes. Additionally, although most patients with nodal PTCL are chemotherapy-sensitive, responses are generally not durable and a substantial proportion of patients (between 25% and 56%) will not proceed to an autologous stem cell transplantation (autoSCT) or allogeneic stem cell transplantation (alloSCT) due to early progression or relapse of disease [3]. At present, many physicians adopt autoSCT as upfront consolidation in patients achieving a CR or partial remission (PR) in the following subtypes: PTCL—not otherwise specified (PTCL-NOS), angioimmunoblastic T-cell lymphoma (AITL) and ALCL, ALK-negative. The rationale behind such an approach is discussed below. 

## 2. Methodology

The electronic database MEDLINE (1998–2020) was searched to review the role of HSCT in PTCLs. Titles, keywords and abstracts of citations were reviewed and full copies of potentially suitable articles were assessed further. The following search strategy was used: ‘bone marrow transplantation’ OR ‘hematopoietic stem cell transplantation’ OR ‘stem cell transplantation’ AND ‘lymphoma, t-cell’ OR ‘t-cell, peripheral’ with limits to adults, English language AND Humans.

**Figure 1:**
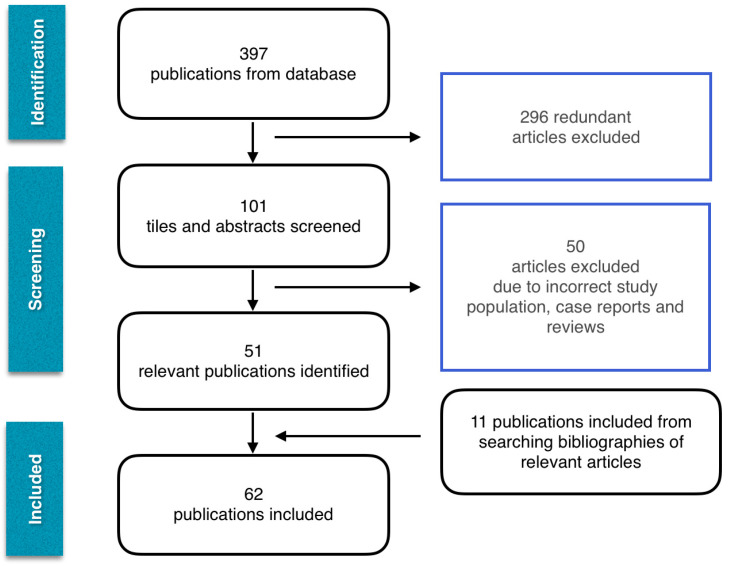
Flow diagram of studies included.

The selection process was undertaken in May 2020. Online search retrieved 397 publications. Only randomised controlled trials, retrospective and prospective studies and meta-analyses were included, resulting in 51 articles. Bibliographies of searched articles were analysed and an additional 11 publications were selected to prevent relevant articles from being missed. The final analysis included 62 articles. A flowchart of the included publications is shown in Figure 1.

Levels of evidence and grades of recommendation have been applied as per the Infectious Diseases Society of America—United States Public Health Service grading system shown in Table 1 [4].

## 3. Autologous Stem Cell Transplantation in Frontline PTCL

### 3.1. Prospective Data

#### Phase II Studies

Corradini et al. reported the combined results of two prospective phase II studies investigating the role of upfront autoSCT in 62 patients with advanced PTCL, including ALK-positive ALCL. Whilst only 74% proceeded to an autoSCT, CR prior to autoSCT was achieved in just 56%. At a median follow-up of 76 months, the estimated 12-year OS, PFS and event-free survival (EFS) were 34%, 55% and 30%, respectively, but, as expected, significantly better outcomes were found in the ALK-positive group as opposed to the non-ALK-positive group (OS 62% versus 21%, *p* = 0.005; EFS 54% versus 18% *p* = 0.006). Multivariate analysis revealed patients attaining CR prior to autoSCT had a statistically significant OS and EFS benefit (*p* < 0.0001). Transplantation-related mortality (TRM) was low at 4.8% [5].

Rodriguez et al. reviewed 26 high-risk PTCL patients, excluding ALK-positive ALCL, where patients were restaged with gallium scans following three cycles of mega-CHOP (cyclophosphamide 2 g/m^2^, doxorubicin 90 mg/m^2^ and vincristine 1.4 mg/m^2^ on day 1, prednisolone 60 mg/m^2^ days 1–5). Patients in CR received another cycle of mega-CHOP and proceeded to an autoSCT whereas gallium scan-positive patients underwent salvage chemotherapy and proceeded to an autoSCT if at least PR was attained. Complete remission was achieved in 46% following the initial three cycles and overall 73% proceeded to transplant achieving an 89% CR rate. After a median follow-up of 35 months, the 3-year predicted OS and PFS were 73% and 53%, respectively. The 2-year predicted OS was 84% and PFS was 56% in the transplant group, indicating that early salvage therapy in patients not attaining a CR may improve clinical outcomes [6].

Mercadal et al. studied 41 PTCL patients who were administered three courses of high-dose CHOP alternated with three courses of etoposide, methylprednisolone, cytarabine, cisplatin (ESHAP) followed by autoSCT if CR or PR were achieved. Only 41% were able to proceed to transplant (*n* = 17). Four-year PFS and OS were 30% and 39%, respectively. The International Prognostic Index (IPI) was noted to be of prognostic value [7].

A German study by Reimer et al. reviewed 83 PTCL patients (ALK-positive ALCL excluded) who received four to six cycles of CHOP followed by autoSCT if at least a PR achieved (*n* = 55, 66%). In an intent-to-treat analysis overall response rates post autoSCT was 66% (65% CR, 8% PR) and with a median follow-up of 33 months. In the CR group the estimated 3-year OS, disease-free survival (DFS) and PFS were 48%, 53% and 36%, respectively. The 3-year OS for patients who underwent autoSCT was 71% compared with only 11% in the non-transplant group [8]. A 5-year follow-up study with the addition of 28 new patients (total *n* = 111) was published in 2016. Complete remission rate after myeloablative therapy was 59%. The estimated 5-year OS, DFS and PFS rates were 44%, 54% and 39%, respectively. The results of this study confirm that upfront autoSCT can result in long-term remissions in patients with all major subtypes of PTCL in CR [9].

The largest and most important prospective study to date which drives current practice was conducted by D’Amore et al. from the Nordic Lymphoma Group (NLG-T-01). They enrolled 166 patients (ALK-positive ALCL excluded) and administered biweekly CHOP and etoposide (CHOEP), etoposide omitted if age > 60 years) followed by autoSCT if at least PR achieved (71%). Following a long follow-up period of 60.5 months, 5-year OS and PFS in a cohort of patients who underwent an autoSCT were 51% (95% CI 43–59) and 44% (95% CI 36–52), respectively. Subtype-specific analysis revealed the highest OS and PFS occurring in patients with ALCL (5 years: OS, 70%; PFS, 61%). Patients with AITL and PTCL-NOS had OS at 5 years of 52% and 47% and PFS of 49% and 38%, respectively. The 5-year OS and PFS for enteropathy-associated T-cell lymphoma (EATL) were 48% and 38%, respectively [3].

Please see Table 2.

### 3.2. Registry Data

The Grupo Español de Linfomas/Trasplante Autólogo de Médula Ósea (GEL-TAMO) registry in Spain analysed 37 patients transplanted in first complete remission (CR1). Notably, the 5-year OS was 80% (95% CI 65–95) with a statistically significant longer survival when compared with patients transplanted in later CRs (*p* = 0.007) [12]. 

The Swedish Lymphoma Registry included 252 nodal PTCL and EATL patients (excluding ALCL, ALK-positive). Whilst 128 cases were planned for an upfront autoSCT, following a median follow-up of 8.1 years, patients in the autoSCT intent-to-treat had a favourable OS (HR 0.57; *p* = 0.005) and PFS (HR 0.59; *p* = 0.006). Five-year PFS and OS were 41% and 48%, respectively, compared with 20% and 26% in the non-transplant group [13]. 

More recently, the Comprehensive Oncology Measures for Peripheral T-cell Lymphoma Treatment (COMPLETE) registry in the United States from 56 academic and community centres (*n* = 499 enrolled patients) reported its outcomes. A prospective multicentre cohort study was published in 2019 and focused on 119 newly diagnosed patients with nodal PTCL (excluding ALCL, ALK-positive) achieving CR1. Whilst 36 patients proceeded to autoSCT, 83 patients received chemotherapy only. Following a median follow-up of 2.8 years, the estimated 2-year OS and PFS rates for CR1 patients were 75.3% (95% CI 67.8–83.7) and 63.4% (95% CI 55.2–72.9) as compared to 41.9% (95% CI 31.6–55.6) and 19.3% (95% CI 11.8–31.8) in patients not achieving CR1. Regardless of autoSCT status, patients with ALCL had a significantly longer OS and PFS. In terms of OS and PFS, there was a trend only in favour of autoSCT for OS (*p* = 0.06 and *p* = 0.23). When PTCL subtypes were considered, interestingly, survival outcomes for patients with AITL appeared more favourable in the autoSCT group with estimated 2-year OS and PFS in CR1 being 93.3% (95% CI 81.5–100) and 68.8% (95% CI 48.4–95.7), respectively, compared with 52.9% (95% CI 33.8–82.9) and 41.2% (95% CI 23.3–72.7), respectively, in the non-autoSCT group. However, definitive conclusions could not be made due to small numbers (*n* = 35). There was no statistically significant OS or PFS benefit for transplant in ALCL (*p* = 0.39, *p* = 0.27) or for patients with PTCL-NOS (*p* = 0.78, *p* = 0.46). Notably, PTCL-NOS was evenly distributed between the transplant and non-transplant groups, whereas AITL and advanced-stage (III/IV) disease (92% versus 64%, *p* < 0.01) were predominantly represented in the autoSCT cohort. In contrast, ALCL was more frequent in the non-autoSCT group [14]. 

Please see Table 3.

### 3.3. Retrospective Data

J. Yin et al. published a meta-analysis investigating the role of autoSCT as a first-line treatment in PTCL. This included a total of 1021 patients from six prospective (five of these mentioned above) and fifteen retrospective studies between 1990 and 2012. According to the pooled results, up-front autoSCT showed a non-statistically significant trend in survival benefit in PTCL patients compared with conventional chemotherapy alone (HR 0.81, 95% CI 0.31–2.13). The CR rate after autoSCT ranged from 51 to 100%, whilst the OS ranged from 58% at 1 year to 34% at 12 years. They concluded that achievement of CR before transplantation was a strong predictor of survival benefit with dismal survival outcomes if patients failed to attain CR before transplantation (*p* = 0.004). Additionally, they suggested a significant survival advantage after transplantation for patients with low IPI (≤2) risk compared to patients with high IPI risk (*p* < 0.0001) based on six studies. Likewise, based on four studies, a low prognostic index for PTCL (PIT) had better outcomes (PIT < 2, HR 0.31 (95% Cl 0.17–0.8); PIT < 3, HR 0.31 (95% Cl 0.18–0.54)) [16].

Data from the MD Anderson Cancer Centre (MDACC) analysed the outcomes of 47 patients who underwent a HSCT in a frontline setting (autoSCT, *n* = 47; alloSCT, *n* = 11) with PTCL-NOS and AITL being the dominant histological subtypes. Four-year OS and PFS for autoSCT were 76% (95% CI 56–88) and 56% (95% CI 43–71), respectively, with no statistically significant difference compared with frontline alloSCT [17].

A large multicentre retrospective review from 14 centres in France, Belgium and Portugal (LYSA centres) analysed the role of upfront autoSCT in 269 patients with PTCL (including ALCL, ALK-positive). Neither the Cox multivariate model (HR = 1.02; 95% CI, 0.69–1.50 for PFS and HR = 1.08; 95% CI 68–1.69 for OS) nor the propensity score analysis after stringent matching for potential confounding factors (logrank *p* = 0.90 and 0.66 for PFS and OS, respectively) found a survival advantage in favour of autoSCT as a consolidation procedure for patients in response after induction over observation. Subgroup analyses did not reveal any further difference for patients according to response status, disease stage or risk category [18]. 

A meta-analysis by El-Asmar et al., published in 2016, investigated the efficacy of autoSCT in PTCL as frontline consolidation and in the relapsed and refractory (RR) setting. This analysis included 27 studies until the year 2015, mostly retrospective in nature, and included a total of 1359 patients. Three prospective (*n* = 179) and 16 retrospective studies (*n* = 599) studies reviewed the efficacy of autoSCT in the frontline setting. Pooled analysis of prospective studies revealed a PFS of 33% (95% CI 14–56) and OS of 54% (95% CI 32–75). Pooled analysis of retrospective studies demonstrated a PFS and OS of 55% (95% CI 40–69) and 68% (95% CI 56–78), respectively. The difference in outcomes was thought to be due to better control of confounding factors in prospective studies. They concluded that autoSCT is a reasonable option in frontline consolidation with resulting OS rates of 54% to 68% and a relatively low TRM ranging from 2% to 6%; however, this is debatable given the PFS from prospective studies was only 33% [19].

Please see Table 4.

## 4. Allogeneic Stem Cell Transplantation in Frontline PTCL

### 4.1. Prospective Data

The first study testing the feasibility and efficacy of alloSCT in upfront PTCL was published by Corradini et al. in 2014; 61 patients (<60 years) were administered alemtuzumab (ALZ) + CHOP, followed by one or two cycles of high-dose methotrexate given as a continuous infusion (HyperCHidam (1.6 g/m^2^) on day 1; cyclophosphamide 300 mg/m^2^ and high-dose cytarabine 2 g/m^2^ every 12 hours for 3 days) and patients achieving a PR or CR (*n* = 38) entered the transplant phase (alloSCT, *n* = 23; autoSCT, *n* = 14). Four-year OS were 92% for autoSCT and 69% for alloSCT (*p* = 0.10) and 4-year PFS were 70% and 69% for those receiving autoSCT or alloSCT (*p* = 0.92), respectively. In a multivariate analysis, the achievement of CR maintained for at least 6 months had a significant effect on PFS and OS (*p* = 0.038, *p* = 0.009). Although no significant difference in survival outcomes were observed despite prolonging DFS in young patients, this study was not designed and powered to evaluate the differences between transplant types [10].

Loirat et al. analysed 49 patients with PTCL where 29 patients proceeded to an upfront alloSCT. For transplanted patients, the 1- and 2-year OS were 76% (95% CI 62–93) and 72.5% (95% CI 58–91), respectively, as opposed to 59% (95% CI 47–75) and 55% (95% CI 43–71) in all patients, including those transplanted. A substantial proportion did not proceed to alloSCT due to disease progression or insufficient response after induction. One-year TRM after alloSCT was only 8.2% (95% CI 0–18.5) and 2-year PFS rate for patients who did not proceed to alloSCT (for medical reasons, refusal or without a human leukocyte antigen-matched donor) was 30% (95% CI 15.5–58.5) [11]. 

Please see Table 2.

### 4.2. Registry Data

Retrospective analysis of the Johns Hopkins bone marrow transplant registry reviewed 44 alloSCT (reduced-intensity conditioning (RIC) *n* = 24; myeloablative conditioning (MAC) *n* = 20) performed over approximately two decades for PTCL. There was a trend toward superior outcomes for alloSCT in CR1 versus beyond first remission, with an estimated 2-year PFS of 53% (95% CI 33–77) versus 29% (95% CI 9–45), *p* = 0.08. Allografts in CR1, including those with primary induction failure, had an estimated 5-year PFS of 54% and an estimated 5-year OS of 54% as well, comparing favourably to the upfront autoSCT outcomes, particularly considering that RIC was used in more than half of alloSCT and that a lower rate of graft versus host disease (GVHD) and non-relapse mortality (NRM) was evident [15]. 

Please see Table 3.

## 5. Autologous Stem Cell Transplantation in Relapsed and Refractory PTCL

### 5.1. Registry Data

The previously mentioned GEL-TAMO registry reviewed 78 patients undergoing an autoSCT between 1990 and 1999, in the salvage setting. Five-year predicted OS, time to treatment failure and DFS were 45% (95% CI 31–59), 39% (95% CI 27–51) and 49% (95% CI 34–64), respectively, with no difference between patients transplanted in PR (5-year OS 46% as opposed to patients transplanted in second or subsequent CR (5-year OS 42%) [12]. 

In 2013, HSCT outcomes of a large cohort of 241 patients collected on the Centre for International Blood and Marrow Transplant Research (CIBMTR) database with ALCL (*n* = 112), PTCL-NOS (*n* = 102), AITL (*n* = 27) were analysed. Allograft and autograft outcomes were compared. There was a substantial difference in baseline characteristics with patients undergoing autoSCT more likely to be in CR1, have chemotherapy-sensitive disease, have ALCL subtype and have fewer prior lines of therapy. Therefore, a multivariate analysis was performed which did not find a significant difference in relapse/progression between autoSCT and alloSCT (*p* = 0.1451), although NRM was significantly higher, 6% compared with 34% in the latter. When specifically examining autoSCT recipients beyond CR1 by histology, patients with ALCL (*n* = 39) had 3-year PFS and OS rates of 50% (95% CI 34–66) and 65% (95% CI 49–80), respectively. Patients with PTCL-NOS (*n* = 28) had 3-year PFS and OS rates of 29% (95% CI 12–50) and 42% (95% CI 22–62), respectively. Whilst only six patients with AITL underwent autoSCT in the relapsed setting, 1-year PFS and OS rates were both 33% (95% CI 5–72). Notably, despite the known impact of ALK status on outcomes of ALCL, this study did not include ALK status in the analysis. However, one should bear in mind that this is a study of relapsed disease, so the included ALK-positive patients would have been the uncommon aggressive subgroup [40].

Please see Table 5.

### 5.2. Retrospective Data

The aforementioned meta-analysis by El-Asmar et al. included 15 retrospective studies (*n* = 581) reported on autoSCT in the RR setting. For RR PTCL, the pooled rates of PFS, OS and relapse/progression were 36% (95% CI 32–40), 47% (95% CI 43–51) and 51% (95% CI 39–62), respectively. Transplantation-related mortality was 10% (95% CI 5–17). They concluded that relapse/progression and TRM were higher when autoSCT was offered in RR disease (relapse/progression 36% and TRM 6% in the frontline setting) [19].

Chen et al. reported an experience at Stanford where 32 RR PTCL patients underwent autoSCT (primary refractory, *n* = 11). Five-year PFS and OS were 12% and 40%, respectively. They indicated a minimal durable benefit with autoSCT and proposed the consideration of novel strategies or alloSCT in the RR setting. This was in contrast to a favourable 5-year PFS of 51% and OS of 76% in CR1 [44].

The previously mentioned team from the MDACC reviewed 76 RR PTCL patients who underwent a HSCT between 1990 and 2009 (autoSCT, *n* = 41; alloSCT, *n* = 35) and demonstrated better outcomes with autoSCT, especially in chemotherapy-sensitive disease, with no survival benefit with alloSCT. Notably, the two cohorts were not comparable, with alloSCT having a younger median age (43 years versus 56 years) and more extra-nodal histologies. The 4-year predicted OS rates were superior in autoSCT compared with alloSCT (50% versus 36%, *p* < 0.05). There was no statistically significant difference in 4-year PFS between the two groups. Additionally, there were no discernible differences in OS amongst the major histological subtypes [17].

Please see Table 6.

## 6. Allogeneic Stem Cell Transplantation in Relapsed/Refractory PTCL

### 6.1. Prospective Data

A small phase II prospective study by Corradini et al. included 17 patients (primary chemotherapy refractory, *n* = 2; relapsed disease, *n* = 15; disease relapse post autoSCT, *n* = 8) who received salvage chemotherapy followed by RIC alloSCT. The estimated 3-year OS and PFS were 81% (95% CI 62–100) and 64% (95% CI 39–89%), respectively. The estimated probability of NRM at 2 years was 6% (95% CI 1–17). Donor lymphocyte infusions induced a response in two patients progressing after alloSCT [56]. 

### 6.2. Registry Data

Retrospective analysis of a French registry included 77 PTCL patients who underwent an alloSCT between 1988 and 2006. The median number of prior lines of therapies was two with 25% of cases having a prior autoSCT. At the time of alloSCT, 31 patients were in CR, whereas 26 were in PR. Five-year TRM was 33% (95% CI 24–46). The 5-year OS and event EFS rates were 57% (95% CI 45–68) and 53% (95% CI 41–64), respectively. In multivariate analysis, chemotherapy resistant disease at the time of alloSCT and the occurrence of severe grade 3–4 acute graft versus host disease (aGVHD) had a negative prognostic value for OS (*p* = 0.0003 and 0.0001, respectively) [41]. 

As described previously, the CIBMTR registry reviewed 126 alloSCT patients in 2013 and noted a 3-year PFS of 31% despite being heavily pre-treated and with refractory disease, but there was no significant difference in relapse/progression between autoSCT and alloSCT (*p* = 0.1451). Regimen intensity, either MAC, non-myeloablative conditioning (NMAC) or RIC, did not have an impact on PFS, OS or NRM. Two or fewer lines of pre-transplantation therapy (HR 5.02; 95% CI 2.15–11.72) were prognostic of survival [40].

The CIBMTR registry also reviewed the outcomes of alloSCT in 249 patients with RR AITL, with a median of three prior lines of therapy, demonstrating durable disease control even in patients who failed autoSCT and patients with refractory disease at the time of alloSCT. The majority had chemotherapy-sensitive disease at time of alloSCT (79%) and received an NMAC/RIC conditioning regimen; grade 3-4 acute graft versus host disease (aGVHD) was 12% (95% CI 8–17), 1-year chronic graft versus host disease (cGVHD) was 49% (95% CI 43–56), 1-year NRM of 19% (95% CI 14–24), 4-year PFS and OS of 47% (95% CI 41–54) and 56% (95% CI 49–63), respectively. Four-year PFS and OS in refractory patients were 38% and 52%, respectively. [42].

Fukano et al. analysed 38 patients (age 3–30 years) with RR ALCL who had undergone an alloSCT (RIC, *n* = 8; MAC, *n* = 30) in a Japanese registry. The 5-year OS rates in the RIC and MAC groups were 100% and 49%, respectively (*p* = 0.018). The 5-year EFS rates were 88% in the RIC and 43% in the MAC group (*p* = 0.039). Whilst four of the eight patients in the RIC cohort showed residual disease at alloSCT, all patients achieved a CR. These data suggest that RIC alloSCT may be effective for RR ALCL in children, adolescents, and young adults, even in non-CR cases [43]. 

Please see Table 5.

### 6.3. Retrospective Data

Jacobsen et al. examined the outcome of 52 patients who underwent an alloSCT at the Dana-Farber Cancer Institute/Brigham and Women’s Hospital and indicated that alloSCT can produce long-term disease control with a notable plateau in OS and PFS curves. A wide variation of PTCL subtypes were included, with the majority being PTCL-NOS (38%) and 79% of patients had undergone a prior autoSCT. Three-year OS was 41% (95% CI 28–55) and 3-year PFS was 30% (95% CI 18–43) with the nodal histology group performing better compared with extranodal histologies. The incidence of GVHD was acceptable (grade III–IV aGVHD 6% and cGVHD 27%). Despite a significantly higher risk of relapse with RIC (*p* = 0.00005) compared with ablative conditioning, there was no difference in OS or PFS [48]. 

Czajczynska et al. reviewed 24 RR PTCL patients with varying subtypes who underwent induction therapy, usually the cladribine, cytosine arabinoside, and etoposide with granulocyte colony-stimulating factor support (CLAEG) protocol, and proceeded immediately to conditioning with carmustine, etoposide, cytarabine, melphalan (BEAM) and medium-dose alemtuzumab. Whilst 50% had never achieved a CR previously, 20 of 22 assessable patients achieved a CR with acceptable toxicity (grade II–IV aGVHD 25% and cGVHD 30%). One-year and 3-year OS were 58.3% (95% CI 40.6–83.8) and 42.4% (95% CI 25.4–70.8), respectively [53].

A retrospective analysis from a Chinese centre in 2016 that included 67 patients with PTCL (autoSCT, *n* = 43; alloSCT, *n* = 24), predominantly PTCL-NOS, did not demonstrate a difference in 5-year PFS (*p* = 0.499) or OS (*p* = 0.566) between alloSCT or autoSCT. However, the two groups were not comparable as patients in CR1 and second CR were predominantly represented in the autoSCT cohort, whilst patients in PR and not in remission were predominantly represented in the alloSCT cohort. Once patients in CR or PR were excluded, alloSCT had a superior 3-year OS (53%, *p* = 0.042) as opposed to patients who underwent an autoSCT, favouring a decision to proceed with alloSCT in patients with primary refractory PTCL [54].

Rohlfing et al. conducted a retrospective single-centre analysis of PTCL patients (ALK-positive patients excluded) referred to the University of Heidelberg, Germany, between 2001 and 2014. This was a two-part study where the first primary objective was to analyse the impact of an ‘intent-to-autoSCT strategy’ in the first-line treatment on outcome and the second primary objective was to investigate the impact of autoSCT and alloSCT on survival after PTCL relapse. Ninety-one of the 117 patients relapsed or progressed (78%) following first-line therapy (40% primary refractory, 16% relapsed within and 21% beyond 6 months). Fifty-one patients (57%) could not proceed to HSCT following re-induction due to progressive disease (38%), poor performance status or advanced age. Of the 38 patients who managed to proceed to HSCT, the majority (31) underwent an alloSCT (seven of them with chemotherapy refractory disease). This study revealed sustained remissions and long-term survival only in patients that underwent an alloSCT with a 5-year predicted OS of 52%, in contrast to all seven patients who received a salvage autoSCT succumbing with a median OS of 10 months [55].

As mentioned above, data from the MDACC demonstrated no survival benefit with alloSCT over autoSCT in RR PTCL [17].

Please see Table 6.

## 7. Comparison of National and Co-Operative Group Guidelines

The National Comprehensive Cancer Network (NCCN) in 2020 [57], American Society for Transplantation and Cellular Therapy (ASTCT) in 2017 [58] and the European Society for Medical Oncology (ESMO) in 2015 [59] all recommend autoSCT as frontline consolidation as an option for nodal PTCL subtypes PTCL-NOS, AITL, and ALK-negative ALCL. 

In ALK-positive ALCL, an upfront autoSCT is not recommended in the ASTCT and NCCN guidelines. Conversely, ESMO further subdivide this category as low and high risk according to the IPI, defined as age (>60 years), performance status (2–4), tumour stage (III–IV), elevated serum lactate dehydrogenase (LDH) and number of extranodal disease sites (>1). Whilst a consolidative autoSCT is not recommended for low-risk disease, they recommend autoSCT in high-risk patients (IPI > 2). All three guidelines recommend autoSCT as frontline consolidation for patients with EATL and consideration of alloSCT in primary refractory or relapsed disease if eligible.

NCCN also suggest that ALK-negative *DUSP22* rearranged cases could be treated similarly to ALK-positive cases without an upfront autoSCT given their favourable prognosis. 

Consideration of upfront alloSCT is suggested by NCCN in patients who do not achieve a PR following induction treatment in nodal PTCL subtypes. Upfront alloSCT is recommended by all for hepatosplenic T-cell lymphoma (HSTCL) if a suitable is donor available. However, some of the panellists in ASTCT favoured frontline autoSCT in advanced age, multiple comorbidities and if CR1.

In relapsed chemotherapy-sensitive disease, ASTCT—using the Grading of Recommendations Assessment, Development and Evaluation (GRADE) methodology—strongly recommend an autoSCT for all nodal PTCL subtypes including ALK-positive ALCL if not done as upfront consolidation or an alloSCT if an autoSCT was performed in the frontline setting. Allogeneic stem cell transplantation is weakly recommended for primary refractory or RR disease. ESMO and NCCN guidelines support this recommendation and favour using a RIC regimen if proceeding with alloSCT.

## 8. Discussion and Recommendations

### 8.1. Why Explore Stem Cell Transplantation in the Management of PTCLs?

Peripheral T-cell lymphomas are a group of lymphomas that portend a poor prognosis with an undefined optimal therapeutic approach. Despite the limited prospective data to clearly demonstrate outcomes following standard CHOP-based therapy, it is recognised that survival outcomes are unsatisfactory, especially compared with B-NHL. Aside from brentuximab vedotin, cyclophosphamide, doxorubicin and prednisolone (A+CHP) for CD30-positive PTCLs and CHOEP for a selected group of patients, intensification of frontline treatment regimens has largely been unsuccessful with benefits mainly being offset by increased toxicity [3,60]. Consolidation with HSCT has thus been introduced in an attempt to reduce disease relapse and achieve durable remissions. Additionally, as shown by Chihara et al. and a report from the International T-cell Lymphoma Project, outcomes of patients failing first-line therapy are dismal and salvage treatment followed by consolidation with HSCT have also been attempted to mitigate such inferior outcomes in the RR setting [61,62].

There is a paucity of literature regarding the effectiveness of alloSCT in PTCL. It does, however, have curative potential mediated by graft versus lymphoma effects. Additional advantages include having a tumour-free graft and the option of donor lymphocyte infusions in the event of recurrent disease. 

### 8.2. Challenges Encountered in Proceeding with Stem Cell Transplantation

The timing and the best modality of autoSCT versus alloSCT in PTCL are controversial. The present body of evidence is based on retrospective studies and registry data with only a handful of prospective studies and no phase III randomised studies. Importantly, these data carry inherent limitations, including small numbers (largest prospective and registry studies having only 166 and 249 patients, respectively), patient selection bias with the selection of patients with chemotherapy-sensitive disease, the marked heterogeneity of PTCL disease subtypes, including variation in the inclusion of favourable histologies such as ALK-positive ALCL, diverse eligibility criteria and differing rates of CR prior to HSCT. Importantly, intent-to-treat analysis is the exception rather than the rule. Indeed, up to 56% of patients are not included due to early relapse or refractory disease hindering progression to transplantation [3]. 

The challenges of proceeding to an alloSCT include the identification of a human leukocyte antigen-compatible donor, the need for a good pre-transplant performance status, organ function and the potentially high TRM, with some studies quoting a risk of up to 33% [41]. Nevertheless, progress over the years in supportive care, GVHD prophylaxis and better selection of conditioning regimens have considerably improved TRM and the availability of alternative stem cell sources such as haploidentical or mismatched donors have increased the access to alloSCT for many patients.

### 8.3. Predictors of Improved Disease Outcomes Following Stem Cell Transplantation

Best results with autoSCT are seen in patients in CR1 at the time of transplantation and this can be challenging as upfront therapies remain inadequate especially in non-nodal aggressive subtypes. This was a unifying observation from the phase II prospective trials where autoSCT was most effective when performed as part of the initial therapy in CR1 and chemotherapy sensitivity was the most important predictor of outcome with 5 year PFS and OS ranging between 39 and 44% and 44 and 51%, respectively [3,9]. Additionally, Rodriguez et al. indicated that early salvage therapy in patients not attaining a CR may improve clinical outcomes with a 2-year predicted OS of 84% and PFS of 56% [6]. The COMPLETE registry also revealed an attractive estimated 2-year PFS and OS rates of 75.3% and 63.4% in patients who underwent an autoSCT in CR1 compared to 41.9% and 19.5% in patients not achieving CR1 [14]. Similarly, disease status at alloSCT is a prognostic indicator [11].

Aside from chemotherapy-sensitive disease, to date, there are no well-defined characteristics that help predict an improved outcome to transplantation. 

### 8.4. Choice of Conditioning Regimen in AlloSCT

Presently, there is no clear evidence to support the use of a specific conditioning regimen. A conditioning regimen has to cautiously balance lymphoma eradication and aid donor engraftment whilst minimising the TRM. The data for a myeloablative conditioning regimen are sparse in this setting and several small retrospective studies have demonstrated that increasing dose intensity with a MAC regimen did not improve outcomes when compared with a RIC regimen [40,41,46,52]. It therefore could be argued that only a graft versus lymphoma effect is responsible for the benefit seen in selected patients, and not dose intensity. A RIC regimen is also attractive as it allows older patients to undergo an alloSCT and its fewer immunosuppressive effects whilst its associated higher risk of relapse can be compensated by lower TRM.

### 8.5. Haematopoietic Stem Cell Transplantation in the Frontline Management of PTCLs

#### 8.5.1. Expected Outcome of PTCL with Standard Frontline Treatment

When considering the upfront management of PTCL, CHOP and CHOP-like regimens, CHOEP, for a selected group of young patients (age < 60, normal LDH), remains the standard of care based on extrapolation from studies conducted on aggressive lymphomas where B and T-cell lymphomas have been grouped together with inadequate statistical power to determine T-cell-specific outcomes. More recently, A+CHP has become the standard of care for CD30-positive PTCL (mainly ALCL). Importantly, expected outcomes with CHOP can be inferred from three studies: the ECHELON-2 data; the recently published study comparing ALZ+CHOP versus CHOP in the elderly (>60 years); and the abstract data on ALZ+CHOP versus CHOP in the young (≤65 years). The ECHELON-2 study demonstrated a 3-year PFS of 44.4% in the placebo + CHOP group (*n* = 226, 70% poor risk ALCL, consolidative autoSCT performed in 17%) with 42% of patients requiring subsequent salvage therapy for residual or progressive disease [60]. The two ALZ+CHOP studies demonstrated a 3-year PFS of 28% and 26%, respectively, for patients receiving CHOP-based therapy [63,64]. 

#### 8.5.2. When Should a Frontline AutoSCT Be Considered?

##### EATL

The evidence for autoSCT as a consolidation strategy is strongest for EATL. This has been demonstrated in the following three prospective studies. The Scotland and Newcastle Lymphoma Group collected data on a total 26 patients who received a novel regimen (one course of CHOP followed by three courses of IVE (ifosfamide, vincristine, etoposide) alternated with intermediate-dose methotrexate) followed by autoSCT if in remission. Five-year PFS and OS were 52% and 60%, respectively, and were significantly improved compared with the historical group treated with anthracycline-based chemotherapy (*p* = 0.01 and *p* = 0.003, respectively) [65]. More recently, a UK phase II study evaluated 21 patients (EATL, *n* = 11) using the induction regimen administered by the Scotland and Newcastle Lymphoma Group. For EATL patients, 1-year OS and PFS were both 45% (95% CI 17–71). Of the five EATL patients receiving autoSCT, only one relapsed [66]. Additionally, the NLG-T-01 study included 21 EATL patients treated with biweekly CHOEP followed by BEAM or BEAC (carmustine, etoposide, cytarabine, cyclophosphamide)-conditioned autoSCT, with a 5-year PFS and OS of 38% and 48%, respectively [3]. A retrospective analysis of the European Society for Bone and Marrow Transplantation (EBMT) registry and a retrospective cohort study both demonstrated a survival advantage with autoSCT [67,68]. Based on these data, autoSCT should be considered as frontline consolidation (Level II, Grade B).

##### PTCL-NOS and AITL

Centred on the data of the six prospective phase II studies and the COMPLETE registry, consolidative autoSCT could be considered for AITL and PTCL-NOS cases in CR1 [3,5,6,7,8,9,14]. This is further supported by two meta-analyses, each including more than 1000 patients; J. Yin et al. demonstrated a non-statistically significant trend in survival benefit with frontline autoSCT and El-Asmar et al. revealed a pooled PFS of 33–55% and an OS of 54–68% with consolidation autoSCT, respectively [16,19]. When analysed by histological subtype, El-Asmar et al. demonstrated a 5-year PFS and OS of 49% and 52% for AITL and 38% and 47% for PTCL-NOS, respectively [19] (Level II, Grade C).

Moreover, two prospective studies have evaluated the role of alloSCT in frontline PTCL [10,11]. These studies did not reveal a significant difference in survival outcomes compared with autoSCT and a substantial proportion of patients unable to proceed with alloSCT due to disease progression or inadequate response. The present data are not sufficient to recommend an alloSCT over autoSCT in the upfront setting, especially in the context of high TRM (Level II, Grade D). 

#### 8.5.3. When Should a Frontline AlloSCT Be Considered?

##### Hepatosplenic T-Cell Lymphoma

The EBMT reported a retrospective registry-based multicentre study of twenty-five patients who were transplanted for HSTCL between 2003 and 2011 (alloSCT *n* = 18, autoSCT *n* = 7). The median interval from diagnosis to transplant was five months and patients had received a median of two (range one to four) lines of prior treatment. Disease status at alloSCT was CR (39%), PR (44%) or refractory disease (17%). With a median follow-up of 36 months, 3-year PFS and OS were 48% and 54% respectively. Of the seven patients who underwent an autoSCT, only one was alive and progression-free 58 months after transplantation [69].

A retrospective systematic review of outcomes in alloSCT reported similar findings. The estimated 3-year relapse-free survival and OS were 42% and 56%, respectively, in 44 patients [70].

Given the dismal prognosis associated with HSTCL and a younger median age of diagnosis, alloSCT can be considered as a consolidative option where feasible, although outcomes remain unsatisfactory with many patients progressing prior to transplantation (Level III, Grade C).

#### 8.5.4. Approaches to Frontline Transplantation in ALCL

##### ALCL, ALK-Negative and ALK-Positive High Risk

The role of transplantation in this setting is controversial. As described previously, a subset of ALCL, ALK-negative cases contain a *DUSP22* translocation with survival rates similar to ALK-positive ALCL and another subset harbour the *TP63* translocation associated with a poorer prognosis, which may assist in deciding on a consolidative approach. To date, no studies have examined these groups separately. 

Several prospective studies suggest a benefit of frontline autoSCT. The previously mentioned NLG-T-01 included 31 ALK-negative ALCL patients with ALK-positive excluded. The ALK-negative group had a 5-year OS and PFS of 70% and 61%, respectively [3]. A sub-analysis of a prospective trial of frontline autoSCT in 202 patients with NHL, which included 15 patients with sALCL (ALK-positive, *n* = 7), was reported by the Groupe Ouest-est d’Etude des Leucémies et Autres Maladies du Sang (GOELAMS) group. All patients entered CR, no relapse occurred and EFS and survival reached 87% with a follow-up of more than five years [71]. The COMPLETE trial included 30 patients with ALCL, ALK-negative and ALK-positive excluded. However, only four patients underwent autoSCT. Regardless of their autoSCT status, patients with ALK-negative ALCL had significantly longer OS than patients with AITL or PTCL NOS and the median PFS was not reached for ALK-negative ALCL, whereas the median PFS times were 35.1 and 46.1 months for AITL and PTCL NOS, respectively. The median PFS was 57.6 months in the autoSCT group, whereas the non-autoSCT group had a median PFS of 47.5 months; however, the difference was not statistically significant [14]. Extrapolating from the above data, it is reasonable to offer autoSCT for patients with ALCL, ALK-negative disease who do not harbour the *DUSP22* rearrangement (Level II, Grade C). The optimum management for those with *DUSP22* rearrangement is unknown but treatment as per ALK-positive disease could be adopted (Level III, Grade C). Patients with a *TP63* rearrangement could be considered for alloSCT (Level III, Grade C).

Similarly, for ALCL, ALK-positive high-risk disease with an IPI of ≥2 (IPI defined according to age (>60 years), performance status (>1), tumour stage (III–IV)), elevated serum LDH and a number of extranodal disease sites (>1), consideration should be given to frontline consolidation with autoSCT (Level II, Grade C). 

##### ALK-Positive Standard Risk

In contrast, due to the favourable survival outcomes with induction therapy alone in ALCL, ALK-positive standard-risk disease (IPI < 2), frontline autoSCT could be omitted, especially if CR1 is achieved (Level II, Grade D). 

##### ALCL, ALK-Negative with *DUSP 22* Rearrangement

Additionally, given the favourable prognosis in ALCL, ALK-negative cases with *DUSP22* translocation, one could argue that frontline autoSCT could be omitted in cases with an IPI < 2, especially if CR1 is achieved (Level III, Grade C).

### 8.6. Haematopoietic Stem Cell Transplantation in Relapsed/Refractory PTCL

#### 8.6.1. Expected Outcome of Peripheral T-Cell Lymphoma with Salvage Treatment

Importantly, as shown by Chihara et al., survival outcomes have not improved since 1999 in subtypes AITL and PTCL-NOS following relapse/progression, despite the introduction of novel agents such as romidepsin and pralatrexate with 5-year OS rates approaching only 10% in patients not undergoing a HSCT [61]. Additionally, a report from the International T-cell Lymphoma Project, which included a total of 1020 patients, has revealed a significant 47% of refractory cases and a 21% relapse rate following frontline treatment with a median time to relapse of only 8 months. Median OS was only 5.8 months; 3-year OS rates were 21% and 28% for refractory and relapsed patients, respectively (*p* < 0.001). Patients receiving a salvage HSCT had a slightly better 3-year survival of 48% compared with 18% for those not proceeding to a HSCT [62]. This underscores an unmet need in the salvage options for the management of PTCL. 

In patients proceeding to transplantation, various salvage combination therapy regimens used in B-cell lymphomas have been investigated with no clear superiority of any single regimen over the other. By way of contrast, for patients not suitable for transplant, standard combination chemotherapy salvage regimens (i.e., ifosfamide, carboplatin, etoposide (ICE), dexamethasone, cisplatin, cytarabine (DHAP), etc.) are not generally recommended as they are toxic and achieve only short remissions. Thus, single-agent regimens such as pralatrexate, romidepsin and gemcitabine should be considered in an attempt to achieve and maintain remissions.

#### 8.6.2. When Should an AutoSCT Be Considered in the Salvage Setting?

##### PTCL-NOS, ALCL (Both ALK-Positive and Negative)

The evidence for autoSCT in the RR setting comes from registry data and retrospective studies. These data suggest that outcomes could be improved if a consolidation HSCT is performed, with the most advantage in the ALCL subtype, improving the 3-year OS to 65% and to approximately 50% in other subtypes. Registry data from CIBMTR revealed no significant difference in survival between autoSCT and alloSCT, albeit a higher TRM of 34% as expected with alloSCT versus 6% [40]. Mixed results have been observed in retrospective studies, with the MDACC data revealing better outcomes with autoSCT and no survival advantage of alloSCT compared with the data from Stanford, revealing poor 5-year PFS and OS rates of 12% and 40% with autoSCT suggesting the need to consider alloSCT or novel strategies [17,44]. Centred on these data, HSCT should be considered for eligible patients in the salvage setting (Level II, Grade C) and chemotherapy-sensitive patients who have not previously had a HSCT preference should be given autoSCT over alloSCT (Level III, Grade C).

In RR ALCL, ALK-negative cases with *DUSP22* translocation and cases with *TP63* rearrangement not suitable for alloSCT, an autoSCT should be performed if this is not done as part of frontline consolidation (Level III, Grade C).

#### 8.6.3. When Should an AlloSCT Be Considered in the Salvage Setting?

##### AITL

Angioimmunoblastic T-cell lymphoma is a somewhat special circumstance where patients are more likely to benefit from an alloSCT in the RR setting compared with other nodal PTCLs based on two retrospective registry data [41,42] (Level II, Grade C).

The CIBMTR registry demonstrated a 4-year PFS and OS of 47% (95% CI 41–54) and 56% (95% CI 49–63) in RR AITL with a median of three prior lines of therapy and a median age of 56 years. Additionally, relapses were noted to plateau at 2 years post alloSCT and provided durable disease control even in patients with a failed prior autoSCT and patients with refractory disease at the time of alloSCT (4-year PFS 38%, OS 52%) [42]. 

The French registry revealed a favourable survival advantage for RR AITL compared with other histological subtypes with a 5-year OS and EFS rates of 80% (95% CI 39–94) and 80% (95% CI 39–94) as opposed to 63% (95% CI 41–79) and 58% (95% CI 35–75) for PTCL patients, 55% (95% CI 35–72), and 48% (95% CI 28–65) for ALCL patients, respectively. Nevertheless, univariate analysis showed no statistical difference in OS, EFS and TRM according to the histological subtype [41].

### 8.7. Future Directions

Given its extreme heterogeneity and rarity, conducting well designed prospective RCTs addressing areas of unmet need, especially in specific PTCL subtypes with inherently varied clinical behaviours, is a formidable task but needs to be undertaken. In an era where novel agents are emerging as potential frontline options, it is largely unknown if these targeted therapies are able to achieve durable remissions. Such an approach may eventually obviate the need for HSCT, particularly in frontline settings, similar to our current practice in B-NHLs. 

Disease rarity and heterogeneity add to the complexity of managing PTCL. Excitingly, gene expression profiling has recently improved our diagnostic and prognostic ability and it is becoming increasingly clear that PTCL is not one disease, but many biologically distinct subsets with varying prognostic implications and outcomes, especially in ALCL, ALK-negative and PTCL-NOS subtypes. To date, these subtypes have not been explored individually. This underscores the importance of enrolling patients in clinical trials or collaborating to form larger registries so specific subtypes can be analysed individually. 

## 9. Conclusions

On the basis of currently available data, HSCT remains an important therapeutic modality in the management of PTCLs in both the frontline and salvage settings, however the lack of good quality randomized data to guide management has resulted in lack of consensus and occasionally conflicting recommendations in the literature. The rapidly evolving changes in the diagnostic and therapeutic landscape, will necessitate an ongoing evaluation of the role of HSCT in the management of these heterogeneous diseases.

## Figures and Tables

**Table 1:** Levels of evidence and grades of recommendation.

**Levels of evidence**	
I	Evidence from at least one properly randomized, controlled trial
II	Evidence from at least one well designed clinical trial without randomization, from cohort or case-control analytic studies, from multiple time-series studies or from dramatic results in uncontrolled experiments
III	Evidence from opinions or respected authorities, based on clinical experience, descriptive studies or reports of expert committees
**Grades of recommendation**	
A	Good evidence to support a recommendation for use
B	Moderate evidence to support a recommendation for use
C	Poor evidence to support recommendation
D	Moderate evidence to support a recommendation against use
E	Good evidence to support a recommendation against use

Adapted from Infectious Diseases Society of America—United States Public Health Service grading system [4].

**Table 2:** Autologous and allogeneic stem cell transplantation in the frontline setting: prospective studies.

Study	Year	Cases (*n*)	PTCL Subtypes (*n* = ALCL)	ITT Analysis (*n* = Failed to Proceed to HSCT)	% Proceeded to HSCT	Response Prior to AutoSCT	Survival	TRM %
**Autologous Stem Cell Transplantation**								
Corradini et al. [5]	2006	62	All subtypes including ALCL (ALK+ *n* = 19 ALK− *n* = 4)	Yes (*n* = 16)	74	CR: 66% PR: 16%	DFS: 55% at 12 years OS: 30% at 12 years	4.8
Rodgriguez et al. [6]	2007	26	All subtypes including ALCL (ALK− *n* = 8)	No (*n* = 7)	73	CR: 65% PR: 8%	PFS: 53% at 3 years OS: 73% at 3 years	0
Mercadal et al. [7]	2008	41	All subtypes including ALCL (ALK+ *n* = 1)	No (*n* = 7)	41	CR: 49% PR: 10%	PFS: 30% at 4 years OS: 39% at 4 years	-
Reimer et al. [8]	2009	83	All subtypes including ALCL (ALK− *n* = 13)	Yes (*n* = 28)	66	CR: 39% PR: 40%	OS: 48% at 3 years	3.6
d’Amore et al. [3]	2012	166	All subtypes excluding ALCL, ALK+ (ALK− *n* = 31)	Yes (*n* = 51)	72	CR: 51% PR: 30%	PFS: 44% at 5 years OS: 51% at 5 years	4
Wilhelm et al. [9]	2016	111	All subtypes including ALCL (ALK− *n* = 16)	Yes (*n* = 36)	68	CR: 62% PR: 20%	PFS: 39% at 5 years OS: 44% at 5 years	3.6
**Allogeneic stem cell transplantation**								
Corradini et al. [10]	2014	61	All subtypes excluding ALCL, ALK+ (ALK− *n* = 12)	No (*n* = 24)	alloSCT 38	CR: 87% PR: 13%	PFS: 69% at 4 years OS: 69% at 4 years	13
					autoSCT 23	CR: 71% PR: 29%	PFS: 70% at 4 years OS: 92% at 4 years	
Loirat et al. [11]	2014	49	All subtypes excluding ALCL, ALK+ (ALK− *n* = 7)	Yes (*n* = 13)	60	CR: 41.5% PR: 58.5%	PFS: 62.5% at 2 years OS: 72.5% at 2 years	8.2

PTCL, peripheral T-cell lymphoma; ALCL, anaplastic large-cell lymphoma; *n*, number; ITT, intention-to-treat; TRM, treatment related mortality; CR, complete remission; PR, partial remission; PFS, progression-free survival; OS, overall survival; DFS, disease-free survival; autoSCT, autologous stem cell transplantation; ALCL, anaplastic large-cell lymphoma; ALK+, anaplastic lymphoma kinase-positive; ALK−, anaplastic lymphoma kinase-negative.

**Table 3:** Autologous and allogeneic stem cell transplantation in the frontline setting: registry data.

Autologous Stem Cell Transplantation						
Study	Year	Cases (*n*)	PTCL Subtypes (*n* = ALCL)	Median Follow-Up (Years)	Survival	TRM (%)
Rodriguez et al. [12] (GEL-TAMO)	2003	37	All subtypes including ALCL	37	DFS: 79% at 5 years OS: 80% at 5 years	8
Ellin et al. [13] (Swedish)	2014	128	All subtypes including ALCL (ALK− *n* = 24)	8.1	PFS: 41% at 5 years OS: 48% at 5 years	-
Park et al. [14] (COMPLETE)	2019	119 (autoSCT *n* = 36)	All subtypes including ALCL (ALK− *n* = 42)	2.8	OS: 87.6% at 2 year Improved PFS and OS for AITL PFS: 68.8% at 2 years OS: 93.3% at 2 years	-
**Allogeneic Stem Cell Transplantation**						
Kanakry et al. [15] (Johns Hopkins Hospital)	2013	44 (MAC 20 RIC 24)	All subtypes ALCL, (ALK+ *n* = 2 ALCL, ALK− *n* = 5 ALCL, ALKu *n* = 3)	3.9	PFS: 40% at 2 years OS: 43% at 2 years	MAC 10 RIC 8

PTCL, peripheral T-cell lymphoma; ALCL, anaplastic large-cell lymphoma; *n*, number; HSCT, haemopoietic stem cell transplantation; TRM, treatment related mortality; CR, complete remission; PR, partial remission; PFS, progression-free survival; OS, overall survival; DFS, disease-free survival; autoSCT, autologous stem cell transplantation; ALCL, anaplastic large-cell lymphoma; ALK+, anaplastic lymphoma kinase-positive; ALK−, anaplastic lymphoma kinase-negative; ALKu, anaplastic lymphoma kinase-unknown; MAC, myeloablative conditioning regimen; RIC, reduced intensity conditioning regimen.

**Table 4:** Autologous stem cell transplantation in the frontline setting: retrospective data.

Study	Year	Cases (*n*)	PTCL Subtypes	Median Follow-Up (Months)	Overall Survival
Khal et al. [20]	2002	10	All subtypes	13.3	58% at 1 year
Schetelig et al. [21]	2003	29	AITL	60	44% at 5 years
Rodriguez et al. [12]	2003	35	All subtypes	37.5	37% at 5 years
Jantunen et al. [22]	2004	18	All subtypes	24	63% at 5 years
Feyler et al. [23]	2007	64	All subtypes	37	53% at 3 years
Rodriguez et al. [24]	2007	19	AITL	25	60% at 3 years
Rodriguez et al. [25]	2007	74	All subtypes	67	68% at 5 years
Kyriakou et al. [26]	2008	146	AITL	31	59% at 4 years
Niitsu et al. [27]	2008	10	All subtypes	72	60% at 5 years
Prochazka et al. [28]	2009	18	All subtypes	25.7	71% at 2 years
Yang et al. [29]	2009	64	PTCL-NOS	29.7	53% at 3 years
Numata et al. [30]	2010	39	All subtypes	78	62.2% at 5 years
Prochazka et al. [31]	2011	29	All subtypes	55.1	65% at 2 years
Nademanee et al. [32]	2011	12	All subtypes	33.6	92% at 5 years
Czyz et al. [33]	2012	12	ALCL, ALK+ excluded	53	77.2% at 5 years
Mehta et al. [34]	2013	34	ALCL, ALK+ excluded	4 years	67.4% at 4 years
Gui et al. [35]	2014	18	All subtypes	113.5	89% at 5 years
Beitinjaneh et al. [17]	2015	47	All subtypes	35	76% at 4 years
Gritti et al. [36]	2015	21	All subtypes	5.61 years	82% at 3 years
Yam et al. [37]	2016	20	ALCL, ALK+ excluded	23.1	72% at 3 years
Han et al. [38]	2017	52	All subtypes	34	71.1% at 5 years
Wu et al. [39]	2018	47	All subtypes	23.6	89.8% at 2 years
Fossard et al. [18]	2018	269	All subtypes	4.5 years	59.2% at 5 years

PTCL, peripheral T-cell lymphoma; AITL, angioimmunoblastic T-cell lymphoma; PTCL-NOS, peripheral T-cell lymphoma, not otherwise specified; ALCL, anaplastic large-cell lymphoma; ALK+, anaplastic lymphoma kinase-positive; *n*, number.

**Table 5:** Autologous and allogeneic stem cell transplantation in the relapsed and refractory setting: Registry data.

Autologous Stem Cell Transplantation						
Study	Year	Cases (*n*)	PTCL Subtypes (*n* = ALCL)	Median Follow-Up (Months)	Survival	TRM (%)
Rodriguez et al. [12] (GEL-TAMO)	2003	78	All subtypes including ALCL	37	DFS: 49% at 5 years OS: 45% at 5 years	8 (37 months)
Smith et al. [40] (CIBMTR)	2013	75	All subtypes including ALCL (*n* = 39)	73	PFS: 41% at 3 years OS: 53% at 3 years	6 (3 year)
**Allogeneic stem cell transplantation**						
Le Gouill et al. [41] (French)	2008	77	All subtypes including ALCL (*n* = 27)	43	EFS: 53% at 5 years OS: 57% at 5 years	32 (1 year)
Smith et al. [40] (CIBMTR)	2013	126	All subtypes including ALCL (*n* = 51)	49	PFS: 47% at 3 years OS: 46% at 3 years	34 (3 year)
Epperla et al. [42] (CIBMTR)	2019	249	AITL	49	PFS: 49% at 4 years OS: 56% at 4 years	19 (1 year)
Fukano et al. [43] (Japanese)	2019	38 (RIC 8 MAC 30)	ALCL	72	OS: at 5 years RIC 100%, MAC 49% EFS: at 5 years RIC 88%, MAC 43%	RIC 0 MAC 25.9 (5 year)

PTCL, peripheral T-cell lymphoma; *n*, number; ALCL, anaplastic large-cell lymphoma; TRM, treatment related mortality; DFS, disease-free survival; OS, overall survival; EFS, event-free survival; PFS, progression-free survival; AITL, angioimmunoblastic T-cell lymphoma; RIC, reduced-intensity conditioning; MAC, myeloablative conditioning.

**Table 6:** Autologous and allogeneic stem cell transplantation in the relapsed and refractory setting: Retrospective studies.

Autologous Stem Cell Transplantation						
Study	Year	Cases (*n*)	PTCL Subtypes (*n* = ALCL)	Median Follow-Up (Months)	Survival	TRM (%)
Kewalramani et al. [45]	2006	24	All subtypes excluding ALCL, ALK+ (ALCL, ALK− *n* = 4)	6 years	PFS: 24% at 5 years OS: 33% at 5 years	-
Chen et al. [44]	2008	32	All subtypes including ALCL (*n* = 13)	-	PFS: 12% at 5 years OS: 40% at 5 years	4
Nademanee et al. [32]	2011	55	All subtypes including ALCL, ALK+	33.6	PFS: 32% at 5 years OS: 45% at 5 years	0
Czyz et al. [33]	2012	46	All subtypes including ALCL, ALK+	53	PFS: 51.7% at 5 years OS: 56.8% at 5 years	-
Beitinjaneh et al. [17]	2015	76 autoSCT 41 alloSCT 35	All subtypes excluding ALCL, ALK+ (ALCL, ALK− *n* = 28)	-	autoSCT OS: 50% at 4 years alloSCT OS: 36% at 4 years PFS: NSD	-
El-Asma et al. [19] (meta-analysis)	2016	581	All subtypes including ALCL	-	PFS: 36% OS: 47%	10
Wu et al. [39]	2018	32	All subtypes including ALCL	23.6	PFS: 32.9% at 2 years OS: 50.5% at 2 years	-
**Allogeneic Stem Cell Transplantation**						
Kyriakou et al. [46]	2009	45	AITL	29	PFS: 54% at 3 years OS: 64% at 3 years	25% (1 year)
Shustov et al. [47]	2020	17	All subtypes including ALCL	3.3 years	PFS: 53% at 3 years OS: 59% at 3 years	19% (3 year)
Jacobsen et al. [48]	2011	52	All subtypes including ALCL	49	Nodal PTCL PFS: 45% at 3 years OS: 52% at 3 years Extranodal PTCL PFS: 6% at 3 years OS: 23% at 3 years	27 (3 year)
Zain et al. [49]	2011	37	All subtypes including ALCL and CTCL (ALCL *n* = 6)	20.3	PFS: 46.7% at 5years OS: 52.2% at 5 years	28.9% (5 years)
Delioukina et al. [50]	2012	27	All subtypes including ALCL and CTCL	36	PFS: 47% at 2 years OS: 55% at 2 years	22% (2 years)
Dodero et al. [51]	2012	52	All subtypes including ALCL (*n* = 11)	67	PFS: 40% at 5years OS: 50% at 5 years	12% (5 years)
Golberg et al. [52]	2012	34 (*n* = CR1)	All subtypes including ALCL (*n* = 8)	45	PFS: 50% at 2 years OS: 61% at 2 years	18
Czajczynska et al. [53]	2013	24	All subtypes including ALCL (*n* = 4)	44.8	OS 42.4% t 3 years	25
Huang et al. [54]	2017	67 (autoSCT 43 alloSCT 24)	All subtypes excluding ALCL, ALK+ (ALCL, ALK− *n* = 19)	27	autoSCT PFS: 49% at 5 years OS: 57% at 5 years alloSCT PFS: 54% at 5 years OS: 55% at 5 years	18 (alloSCT) 7 (autoSCT)
Rohlfing et al. [55]	2018	117 (RR 91) Underwent SCT *n* = 38 (autoSCT 7 alloSCT 31)	All subtypes including ALCL (*n* = 27)	5.8 years	autoSCT OS: 10 months alloSCT OS: 52% at 5 years	23 (alloSCT)

PTCL, peripheral T-cell lymphoma; *n*, number; ALCL, anaplastic large-cell lymphoma; AITL, angioimmunoblastic T-cell lymphoma; TRM, treatment related mortality; PFS, progression-free survival; OS, overall survival; NSD, not statistically different; autoSCT, autologous stem cell transplantation; alloSCT, allogeneic stem cell transplantation; ALCL, anaplastic large-cell lymphoma; ALK+, anaplastic lymphoma kinase-positive; ALK−, anaplastic lymphoma kinase-negative; RR, relapsed and refractory.

## Abbreviations

**Abbreviation**	**Explanation**
A+CHP	Brentuximab vedotin, cyclophosphamide, doxorubicin, prednisolone
AITL	Angioimmunoblastic T-cell lymphoma
aGVHD	Acute graft versus host disease
ALCL	Anaplastic large-cell lymphoma
ALK	Anaplastic lymphoma kinase
AlloSCT	Allogeneic stem cell transplantation
ALZ+CHOP	Alemtuzumab, cyclophosphamide, doxorubicin, vincristine, prednisolone
ASTCT	American Society for Transplantation and Cellular Therapy
AutoSCT	Autologous stem cell transplantation
BEAC	Carmustine, etoposide, cytarabine, cyclophosphamide
BEAM	Carmustine, etoposide, cytarabine, melphalan
B-NHL	B-cell non-Hodgkin lymphoma
cGVHD	Chronic graft versus host disease
CHOEP	Cyclophosphamide, doxorubicin, vincristine, etoposide, prednisolone
CHOP	Cyclophosphamide, doxorubicin, vincristine, prednisolone
CIBMTR	Centre for International Blood and Marrow Transplant Research
CLAEG	Cladribine, cytosine arabinoside, and etoposide with granulocyte colony-stimulating factor support
COMPLETE	Comprehensive Oncology Measures for Peripheral T-cell Lymphoma Treatment
CR	Complete remission
CR1	First complete remission
DFS	Disease-free survival
DHAP	Dexamethasone, cisplatin, cytarabine
EATL	Enteropathy associated T-cell lymphoma
EBMT	European Society for Bone and Marrow Transplantation
EFS	Event-free survival
ESHAP	Etoposide, methylprednisolone, cytarabine, cisplatin
ESMO	European Society for Medical Oncology
GEL-TAMO	Grupo Español de Linfomas/Trasplante Autólogo de Médula Ósea
GOELAMS	Groupe Ouest-est d’Etude des Leucémies et Autres Maladies du Sang
GVHD	Graft versus host disease
HSCT	Haematopoietic stem cell transplantation
HSTCL	Hepatosplenic T-cell lymphoma
HyperCHidam	High-dose methotrexate given as a continuous infusion (1.6g/m^2^) on day 1; cyclophosphamide 300mg/m^2^ and high-dose cytarabine 2 g/m^2^ every 12 hours for 3 days
ICE	Ifosfamide, carboplatin, etoposide
IPI	International prognostic index
IVE	Ifosfamide, vincristine, etoposide
LDH	Lactate dehydrogenase
MAC	Myeloablative conditioning
MDACC	MD Anderson Cancer Centre
Mega-CHOP	Cyclophosphamide 2 g/m^2^, doxorubicin 90 mg/m^2^ and vincristine 1.4 mg/m^2^ on day 1, prednisolone 60mg/m^2^ days 1–5
NCCN	National Comprehensive Cancer Network
NLG-T-01	Nordic Lymphoma Group-T-01
NMAC	Non-myeloablative conditioning
NRM	Non-relapse mortality
OS	Overall survival
PFS	Progression-free survival
PIT	Prognostic index for peripheral T-cell lymphoma
PR	Partial remission
PTCL	Peripheral T-cell lymphoma
PTCL-NOS	Peripheral T-cell lymphoma-not otherwise specified
RIC	Reduced-intensity conditioning
RR	Relapsed and refractory
TRM	Transplantation-related mortality
